# PROF. DR. EDMUNDO MACHADO FERRAZ: FORMER PRESIDENT OF THE BRAZILIAN COLLEGE OF DIGESTIVE SURGERY

**DOI:** 10.1590/0102-672020220002e1680

**Published:** 2022-09-09

**Affiliations:** 1 Universidade Federal de Pernambuco, University Hospital, Department of Surgery, GeneralSurgery Service - Recife (PE), Brazil.

*“Writing about Prof Edmundo Machado Ferraz is a rather difficult task because in addition to mixing academic and professional facts, it is inevitable, as his son, not to incorporate personal and family interpretations. There were so many occasions in which he acted in a remarkable way that I will surely forget so many other important episodes of his life, which, like great friends that we were, surprised me then and surprise me to this day”.*

**Figure:**
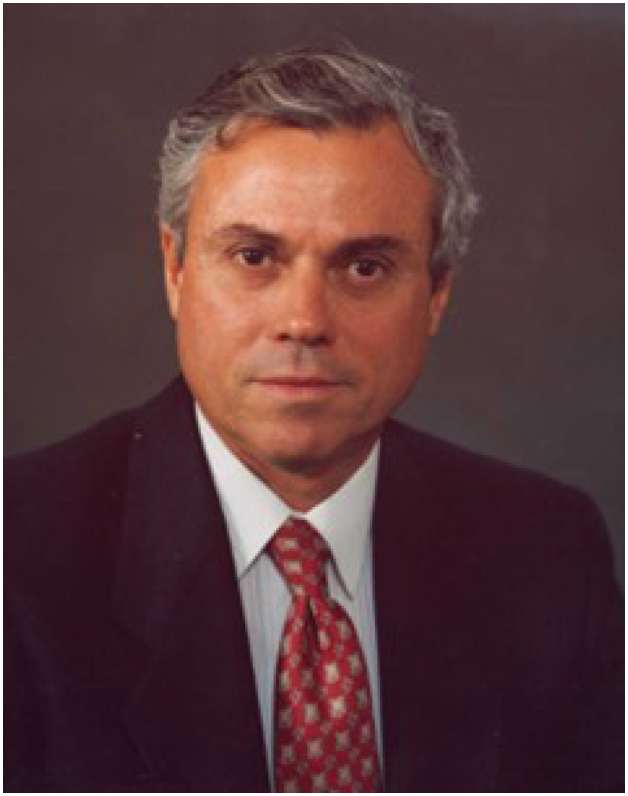


Professor Edmundo lived all his moments intensely. He was not a man of mincing words; he would say what he felt and what he wanted, but always at the right time and in a fair, polite, and caring way.

People who do not take a stand, those who are reluctant, and those who wish to be okay with everything and everyone will certainly make few enemies, but they will also have few admirers, few friends, and, above all, very few followers. They will not form leaders.

More than students, doctors, and surgeons, Professor Edmundo formed eager citizens: citizens who seek better working conditions; citizens who are not satisfied with poor quality medicine, provided by uncommitted people; and citizens who do not accept to offer different treatment to public patients compared to private ones. He formed citizens who protest by working and setting examples; citizens who, in times of conflict, work twice as hard, so that the neediest population is not penalized; and citizens who do not resign and who propose changes. This is the profile of Professor Edmundo’s disciples.

He lived his professional life intensely. He studied cutting-edge Medicine at a public hospital in the Brazilian Northeast. His dedication, commitment, and care for patients who would come to the University Hospital of Universidade Federal de Pernambuco were with a true unanimity of work and seriousness. He would always say: “*Be the first to arrive and the last to leave. If you do that, you will earn the admiration and respect of everyone*.”

Few public servants had the dedication that Professor Edmundo showed throughout his university life. I followed this dedication closely. For over 20 years, I assisted in all the private surgeries performed by him, always after 7 p.m. There were countless times that we left the surgery in a private hospital and went straight to the University Hospital, as the surgery lasted until dawn. Nothing, absolutely nothing, could compromise Professor Edmundo’s work schedule at the University Hospital.

He graduated in Medicine from the Federal University of Pernambuco in 1963, having defended his Doctoral Thesis in Surgery in 1971; soon after, he was approved in a public exam for Associate Professor in 1975. In the same year, he did a Post-Doctoral internship, as a British Council Scholar, at Guy’s Hospital, University of London, England.

He was approved in 17 public exams in his academic career, including those of Full Professor of the Discipline of Surgical Technique in 1987, and of Full Professor of the Discipline of Surgery of the Digestive System in 1990.

He conceived and implemented the 5-year residency in Surgery of the Digestive System. He founded and coordinated the Postgraduate Course in Doctorate in Medicine in 1989 at the Universidade Federal de Pernambuco. He deployed the ATLS program in the northeast region.

He started laparoscopic surgery at the University Hospital, Recife, Pernambuco (with his own resources) in 1993 and implemented the Liver Transplant Program at the University in 1998.

In 1993, he was elected as the President of the Brazilian College of Digestive Surgery (CBCD) by acclamation - the first President outside of São Paulo.

In 1997, he was elected as the President of the Congress of the Brazilian College of Surgeons, held for the first time outside the Rio-São Paulo axis, in Recife, Pernambuco. In 2008, he was elected as the first President outside the Rio-Sao Paulo axis of the Brazilian College of Surgeons (CBC).

He was the founder of the Brazilian Society of Bariatric and Metabolic Surgery, and also founded the Surgical Infection Society — Latin America. Next, he was a consultant at the World Health Organization (WHO) on Infection Control, Antibiotic Use, and Safe Surgery.

His academic life was intense. He was an editor of more than 15 books, wrote over 170 book chapters, and published more than 180 scientific articles^1,2^. He passed away on September 20, 2017.

However, work was only a part of his life. He lived his family life intensely, with my mother, with me, with my brothers, daughters-in-law, and grandchildren.

One day, when he was honored at the Scientific Initiation Meeting of Universidade Federal de Pernambuco, and speaking to younger students, he focused on the desperate search of some for a certain quality of life: “ *Many think that quality of life is to work less and to have more time for leisure*”. He emphasized at that moment that “ *those who do what they like, they have quality of life. When you don’t do what you like, all the time in the world will be too little to rest and relax*.”

I cannot help but be grateful for having lived with my father for 51 years. More than enough time to have his ideas, principles, and ideals settled in me. I have no right to complain. I wanted more. All your disciples want it, but I have to be thankful for every moment we spent together.

On March 1, 1983, when I started my Medicine course at the School of Medical Sciences, today Universidade de Pernambuco, in the morning of the Inaugural Class I received from Professor Edmundo, then Professor of Surgery at Universidade Federal de Pernambuco, my first medicine book, authored by himself, the Manual of Infection Control in Surgery, with the following dedication:

*On the day that you, at age 17, begin your medical course, I wished this was your first medical book. Throughout your course you will learn the Hippocratic teaching that:*

- *art is long*
- *life is short*
- *opportunities are fleeting*
- *experience fails*
- *and judging is difficult.*

*You will see that the path you chose for yourself is very hard and long, but that no profession can be as rewarding as the one you chose and that, through it and your personal fulfillment, you can reach the purpose of your existence: happiness. May life give you everything you rightfully acquire.*
